# Reversible Thrombocytopenia Associated With Rosuvastatin Use: A Case Report

**DOI:** 10.7759/cureus.103224

**Published:** 2026-02-08

**Authors:** Rida Mobeen, Amen Mobeen

**Affiliations:** 1 Medicine, Al-Nafees Medical College, Isra University, Islamabad, PAK; 2 Internal Medicine, Staten Island University Hospital, New York, USA

**Keywords:** drug-induced itp, dyslipidemia, rosuvastatin, south asian, thrombocytopenia

## Abstract

Statins are a class of drugs that act by inhibiting HMG-CoA reductase (3-hydroxy-3-methyl-glutaryl-coenzyme A reductase), an enzyme involved in cholesterol synthesis. As a result, their main uses include the treatment of dyslipidemia and the primary and secondary prevention of coronary artery disease and stroke. Commonly reported side effects include myalgias, transaminitis, and elevated creatine kinase levels. We report a case of a 57-year-old South Asian female who was incidentally found to have thrombocytopenia with no identifiable cause based on her history, physical examination, laboratory evaluation, and imaging. The patient’s medication list was limited to rosuvastatin and calcium supplements, with rosuvastatin being the most likely causative agent. An extensive literature review revealed an association between statin use and thrombocytopenia in general; however, no prior data demonstrated this adverse effect specifically with rosuvastatin. The patient was advised to discontinue rosuvastatin, and her platelet count returned to baseline within three to six months. This report highlights rosuvastatin as a potential cause of reversible drug-induced thrombocytopenia. Further research is required to elucidate the underlying mechanism and confirm this association.

## Introduction

Statins have been in use for several decades [1]. Their use in the secondary prevention of heart disease, peripheral vascular disease, dyslipidemia, and stroke is very well known [2]. Several trials have demonstrated the benefit of statins in the primary prevention of vascular-related deaths, including ischemic heart disease and first stroke [3]. Common side effects include myalgias, elevated liver enzymes, abdominal pain, constipation, dyspepsia, hepatitis, and increased creatine kinase levels [4-5]. Rare side effects include myopathy, rash, sleep disturbances, insomnia, and peripheral neuropathy [6]. The marked increase in statin use has resulted in the identification of additional post-marketing adverse effects [7]. Thrombocytopenia is a rarely reported adverse effect of statin use [6]. The mechanism of drug-induced thrombocytopenia suggests that weakly reactive platelet autoantibodies acquire increased affinity for platelet glycoprotein epitopes in the presence of a sensitizing drug [8]. Clinical manifestations include easy bruising, petechiae, and epistaxis [4]. We report a case of reversible thrombocytopenia in a middle-aged woman. To the best of our knowledge, this is among the few cases reported in the literature.

## Case presentation

A 57-year-old South Asian female with a past medical history of dyslipidemia, cesarean section in 2001, and appendectomy in 2007, was seen in the outpatient clinic for routine follow-up. Complete blood count (CBC) revealed a platelet count of 99,000/mm³, down from 129,000/mm³. About four years ago, during routine examination and laboratory testing, the patient was found to have low platelets, which at the time was attributed to seasonal viral infections. Prior CBCs over the last several months showed fluctuating platelet counts ranging from 112,000 to 160,000/mm³. She denied hematuria, post-menopausal bleeding, or epistaxis. Vital signs were within normal limits, and physical examination revealed no petechiae, ecchymoses, bruising, lymphadenopathy, organomegaly, joint disease, or skin abnormalities. Coagulation profile, renal function, and liver function tests were all within normal limits. Infectious workup, including hepatitis B and C serologies, was negative. Autoimmune testing and inflammatory markers, including C-reactive protein (CRP), erythrocyte sedimentation rate (ESR), anti-nuclear antibody (ANA), and rheumatoid factor, were all unremarkable.

Review of medications revealed rosuvastatin and calcium supplements. The patient had been on atorvastatin since 2000, which was switched to rosuvastatin approximately eight years ago. She was referred to hematology for further evaluation. Peripheral blood smear demonstrated giant platelets, and ultrasound of the abdomen and pelvis showed no abnormalities. The hematologist made a possible diagnosis of immune thrombocytopenia (ITP) and recommended periodic CBC monitoring to trend platelet counts, without any active intervention, since platelets remained above 30,000/mm³ and there were no signs of bleeding.

The patient continued to have CBCs every three to six months to monitor platelet counts. Repeat CBC after one month showed a further decline to 55,000/mm³, prompting the primary doctor to investigate potential causes more thoroughly. On subsequent follow-up, it was considered that rosuvastatin might be contributing to the thrombocytopenia. The patient was therefore advised to discontinue rosuvastatin for a few months. Notably, her CBC after four weeks demonstrated improving platelet counts, and after three months off rosuvastatin, her platelet counts fully recovered and remained persistently within the normal range. The patient was permanently taken off statins and initiated on ezetimibe for management of dyslipidemia. A timeline of the patient’s platelet count trends is presented in Table 1.

**Table 1:** Platelet counts from 2020 to 2024 ^*^CBC was repeated to ensure the platelet count was accurate CBC: complete blood count

Date	Platelet count in thousands per mm^3^ (normal range: 145,000-350,000)
Feb-20	226
Apr-20	133
Jun-20	206
Nov-20	140
Mar-21	334
Jan-22	167
Oct-22	114
Nov-22	112
Dec-22	122
Aug-23	120
Oct-23	129
Dec-23	99
Jan-24	55
Jan-24*	55
Feb-24	144
Mar-24	215
Apr-24	313
May-24	237

## Discussion

In recent years, rosuvastatin and other statins have been widely prescribed, making it easy to overlook potential side effects, including drug-induced thrombocytopenia, which can be caused by a variety of medications [6]. The pathophysiologic mechanisms of drug-induced thrombocytopenia can be broadly categorized into two main types: decreased platelet production due to bone marrow suppression and peripheral platelet destruction, often mediated by immune mechanisms [6]. Although this is a rare adverse effect of statin therapy, our case highlights rosuvastatin-induced thrombocytopenia, which is, to our knowledge, only the second case reported in the literature.

The first case was published in July 2010 [6]. In both cases, the patients had taken the medication to manage high LDL levels, and subsequent CBCs revealed a significant decline in platelet counts over time. In the previous case, although the peripheral blood smear confirmed thrombocytopenia, no additional abnormalities were reported. This contrasts with our case, where giant platelets were observed on the smear, leading to a diagnosis of idiopathic thrombocytopenia. Despite this difference, therapeutic intervention in both cases involved discontinuation of the statin, which was sufficient to restore platelet counts to normal.

Thrombocytopenia has rarely been reported with other statins, such as atorvastatin and simvastatin. The World Health Organization-Uppsala Monitoring Centre (WHO-UMC) scale categorizes a drug-induced adverse effect based on the following criteria: temporal relationship between drug use and the adverse event, absence of other plausible causes, response to drug withdrawal or dose reduction (de-challenge), and response to drug re-introduction (re-challenge) [9]. Our case meets three of the four criteria for a drug-induced adverse effect, specifically thrombocytopenia.

## Conclusions

This report highlights rosuvastatin as a potential cause of reversible thrombocytopenia. Although this side effect is rare with statins overall, there is very limited literature directly associating it with rosuvastatin. Further research is needed to better understand this relationship and to inform its integration into clinical practice.
